# Mechanisms of Sulfoxidation and Epoxidation Mediated by Iron(III)-Iodosylbenzene Adduct: Electron-Transfer vs. Oxygen-Transfer Mechanism

**DOI:** 10.3390/molecules28124745

**Published:** 2023-06-13

**Authors:** Patrik Török, Dóra Lakk-Bogáth, József Kaizer

**Affiliations:** Research Group of Bioorganic and Bio-Coordination Chemistry, University of Pannonia, H-8201 Veszprém, Hungary; patriktrk6@gmail.com (P.T.); lakkd@almos.uni-pannon.hu (D.L.-B.)

**Keywords:** non-heme models, iron(III)-iodosylarene complex, sulfoxidation, epoxidation, O-atom transfer, kinetics

## Abstract

The mechanisms of sulfoxidation and epoxidation mediated by previously synthesized and characterized iron(III)-iodosylbenzene adduct, Fe^III^(OIPh) were investigated using para-substituted thioanisole and styrene derivatives as model substrates. Based on detailed kinetic reaction experiments, including the linear free-energy relationships between the relative reaction rates (log*k*_rel_) and the *σ*_p_ (4R-PhSMe) with *ρ* = −0.65 (catalytic) and *ρ* = −1.13 (stoichiometric), we obtained strong evidence that the stoichiometric and catalytic oxidation of thioanisoles mediated by Fe^III^(OIPh) species involves direct oxygen transfer. The small negative slope −2.18 from log *k*_obs_ versus *E*_ox_ for 4R-PhSMe gives further clear evidence for the direct oxygen atom transfer mechanism. On the contrary, with the linear free-energy relationships between the relative reaction rates (log*k*_rel_) and total substituent effect (TE, 4R-PhCHCH_2_) parameters with slope = 0.33 (catalytic) and 2.02 (stoichiometric), the stoichiometric and catalytic epoxidation of styrenes takes place through a nonconcerted electron transfer (ET) mechanism, including the formation of the radicaloid benzylic radical intermediate in the rate-determining step. On the basis of mechanistic studies, we came to the conclusion that the title iron(III)-iodosylbenzene complex is able to oxygenate sulfides and alkenes before it is transformed into the oxo-iron form by cleavage of the O−I bond.

## 1. Introduction

**Figure 1:**
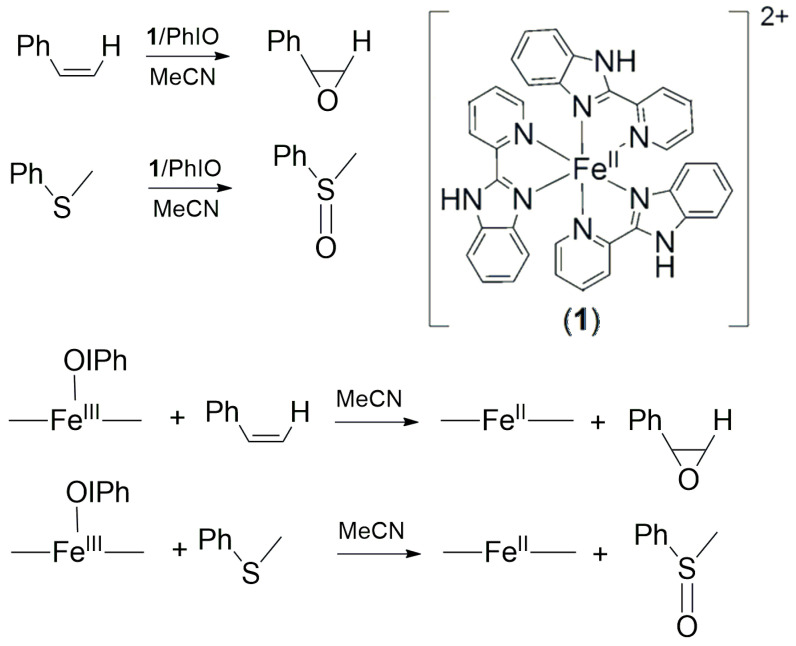
Schematic structure of [Fe^II^(PBI)_3_](CF_3_SO_3_)_2_ (**1**) used in the stoichiometric and catalytic oxidation of thioanisole and styrene.

Iodosylbenzene (PhIO) has attracted considerable attention for its various applications in organic synthesis and biomimetic studies. For example, PhIO has been widely used to produce high-valent metal oxo species, which have been identified as key intermediates in various oxidative reactions in biological systems [1,2,3,4,5,6,7,8,9,10,11,12]. Despite the widespread interest in oxoiron(IV)-mediated epoxidations and sulfoxidations [13,14], relatively little information is available on the mechanism of alkene and sulfide oxidation by synthetic non-heme iron-iodosylbenzene complexes. This is mainly a result of the low stability of the iron-iodosylbenzene intermediates, which prevented direct kinetic measurements of their reactions at ambient temperature. Recently, metal-iodosylbenzene adducts, [M-OIPh], have been successfully prepared, isolated and characterized by various spectroscopic methods, despite their transient and unstable formation. The structure and reactivity of the iodosylarene adduct of heme and non-heme Mn(III), Mn(IV) and Fe(III) complexes have been thoroughly investigated. Recent studies have shown that the metal-iodosylbenzene adduct itself can play an important role in transition metal-catalyzed oxidation reactions [15,16,17,18,19,20,21,22,23,24,25,26,27]. For some multiple oxidation mechanisms, some of showed better reactivity than the corresponding metal-oxo intermediates. The complex [Fe^II^(PBI)_3_](CF_3_SO_3_)_2_ (**1**) (PBI = 2-(2-pyridyl)benzimidazole) proved suitable for the generation of various intermediates depending on the co-oxidant used (Figure 1). Its reaction with H_2_O_2_ and PhIO results in the formation of *μ*-1,2-peroxo-diiron(III) and iron(III)-iodosylbenzene complexes, respectively [28,29,30]. The spectral properties, reactivity and kinetics of Fe^III^(OIPh) bearing PBI ligands towards cycloketones in nucleophilic Baeyer-Villiger reactions [15], and towards benzaldehydes and triphenylmethane in electrophilic C-H oxygenation reactions, were investigated in detail [27].

Significant progress has been made in the last three decades in the understanding and application of nonheme iron complexes in oxidation catalysis. Several ligand systems have been demonstrated as able to form a reactive iron(III)-iodosylarene moiety. Although this part is less commonly examined than the related high-valent oxo congeners, there is no doubt that the iodosylarene entity is a crucial part of the oxidation catalysis in biomimetic model systems. Hence, understanding its properties and reactivity helps tremendously to understand processes in catalytic oxidations. In parallel, highly active and selective catalysts for epoxidation and sulfoxidation have been designed where this moiety is unequivocally involved. To obtain more insight into the mechanisms of the metastable iron(III)-iodosylbenzene mediated sulfoxidation and epoxidation reactions, catalytic and stoichiometric oxidation were carried out using [Fe^II^(PBI)_3_](CF_3_SO_3_)_2_ (**1**) as catalyst and PhIO as oxidant for the catalytic, and the in situ formed Fe^III^(OIPh) species for the stoichiometric oxidation of thioanisole and styrene derivatives (Figure 1).

## 2. Results

### 2.1. Catalytic Sulfoxidation Reaction Mediated by In Situ Generated Iron(III)-Iodosylbenzene Adduct

The catalytic activity of the previously synthesized and characterized ferrous complex, [Fe^II^(PBI)_3_](OTf)_2_ (**1**) was investigated in the oxidation of *para*-substituted thioanisole derivatives, utilizing PhIO as oxidant. The reactions were carried out under standard, optimal catalytic conditions (1:100:300 ratio for catalyst:oxidant:substrate) in acetonitrile at 293 K. A large excess of substrate was used to minimize over-oxidation of the product. It took around 1 h to have about 20–70% yields (based on the oxidant) depending on the substituent on the phenyl ring (Table 1). The iron(II)-catalyzed reactions of thioanisoles produced methyl phenyl sulfoxide derivatives as expected and as a major product in all cases, in addition to one minor product, namely methyl phenyl sulfone derivatives. The effect of electron-donating and electron-withdrawing substituents on the aromatic ring on the relative reactivity has also been studied and showed a significant impact on the catalytic sulfoxidation reaction (Table 1).

**Figure 2:**
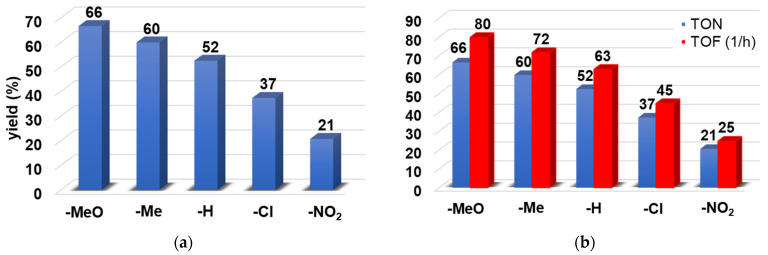
Catalytic oxidation of *para*-substituted thioanisole derivatives carried out by **1** with PhIO as co-oxidant in acetonitrile at 293 K: (**a**) Reaction yields for *para*-substituted thioanisoles. (**b**) TON and TOF values for *para*-substituted thioanisoles. [[Fe^II^(PBI)_3_](OTf)_2_]_0_ = 1 × 10^−3^ M, [PhIO]_0_ = 1 × 10^−1^ M, [4R-PhSMe]_0_ = 3 × 10^−1^ M.

Thioanisols with electron-donating groups, such as -OMe or -Me, on the phenyl ring gave better yields (66% for -OMe and 60% for -Me) than those with electron-withdrawing groups (21% for -NO_2_ and 37% for -Cl) (Figure 2a). The highest TON (turn over number) and TOF (turn over frequency) values can be observed for 4-OMe-PhSMe, at 66 and 80 h^−1^, respectively (Figure 2b).

**Figure 3:**
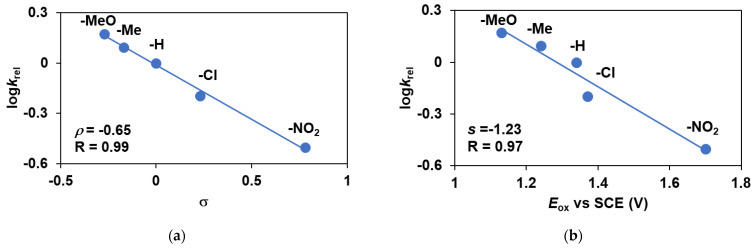
Catalytic oxidation of *para*-substituted thioanisole derivatives carried out by **1** with PhIO as co-oxidant in acetonitrile at 293 K: (**a**) Hammett plot of log*k*_rel_ against the *σ*_p_ of *para*-substituted thioanisoles. (**b**) Plot of log*k*_rel_ against the *E*^0^_ox_ of *para*-substituted thioanisoles. [[Fe^II^(PBI)_3_](OTf)_2_]_0_ = 1 × 10^−3^ M, [PhIO]_0_ = 1 × 10^−1^ M, [4R-PhSMe]_0_ = 3 × 10^−1^ M.

The reactivities of *para*-substituted 4R-PhSMe relative to that of PhSMe were also investigated (Table 1 and Figure 3). It was found that thioanisoles with Hammett treatments of relative reactivities (*k*_rel_ = log(*X*_f_/*X*_i_)/log(*Y*_f_/*Y*_i_), where *X*_i_ and *X*_f_ are the initial and final concentration of 4R-PhSMe, and *Y*_i_ and *Y*_f_ are the initial and final concentration of PhSMe) of various substituents against *σ* gave a *ρ* value of −0.65, which suggests that the behavior of the oxidant generated from **1** and PhIO is mildly electrophilic (Figure 3a). When the log*k*_rel_ values were plotted against the *E*^0^_ox_ potentials of 4R-PhSMe, the plot gave a gradient of −1.23 (Figure 3b). The magnitude of these values may be consistent with a direct O-atom transfer from the substrate in the rate-determining step. Much higher values can be observed for an ET (electron transfer) mechanism [31].

**Figure 4:**
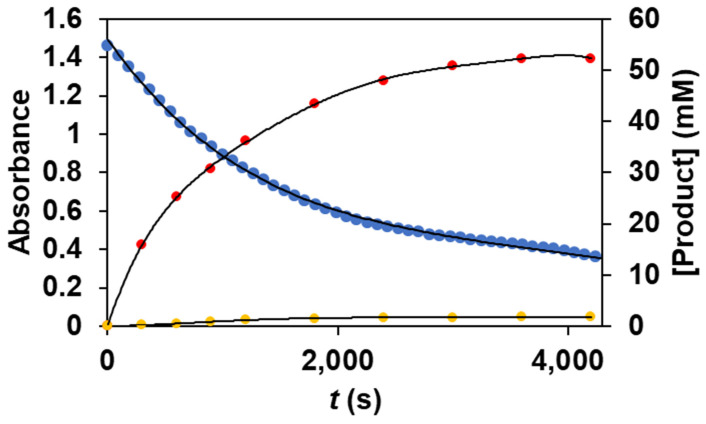
Time course for the catalytic oxidation of thioanisole (•) carried out by **1** with PhIO as co-oxidant in acetonitrile at 293 K, monitored at 760 nm by UV-Vis and by GC, measuring the amounts of the forming phenyl sulfoxide (•) and methyl phenyl sulfone (•). [[Fe^II^(PBI)_3_](OTf)_2_]_0_ = 1 × 10^−3^ M, [PhIO]_0_ = 1 × 10^−1^ M, [PhSMe]_0_ = 3 × 10^−1^ M.

As we noticed before, the reaction of **1** with PhIO results in the formation of reactive Fe^III^(OIPh) species, which suggests that it may have a key role in the catalytic cycles. To prove this assumption, we followed the formation and decomposition of Fe^III^(OIPh) intermediate (the rise and fall of their visible chromophores at λ_max_ = 760 nm, as well as the appearance of the forming products (methyl phenyl sulfoxide and methyl phenyl sulfone) during the oxidation reaction of thioanisole, using parallel UV-Vis and GC measurements). The profile of the catalytic process shows the immediate formation and subsequent decomposition of the spectroscopically characterized Fe^III^(OIPh) species, as well as the formation of the oxidation products that appear parallel to it (Figure 4).

### 2.2. Stoichiometric Sulfoxidation Reaction Mediated by In Situ Generated Iron(III)-Iodosylbenzene Adduct

**Figure 5:**
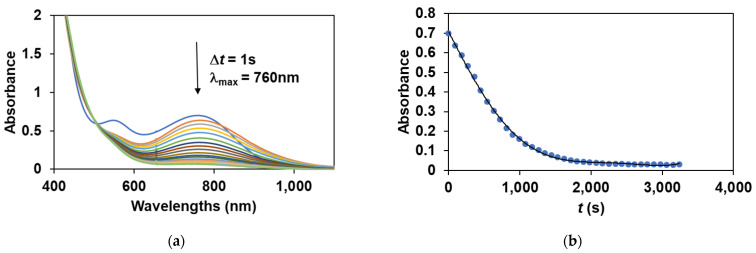
Stoichiometric oxidation of thioanisole with Fe^III^(OIPh) generated in situ by the reaction of **1** with 4 equivalents of PhIO. (**a**) UV-Vis spectral change of Fe^III^(OIPh) (0.4 mM) in the presence of PhSMe (60 mM). (**b**) Time course of the oxidation of PhSMe monitored at 760 nm (**a**).

**Figure 6:**
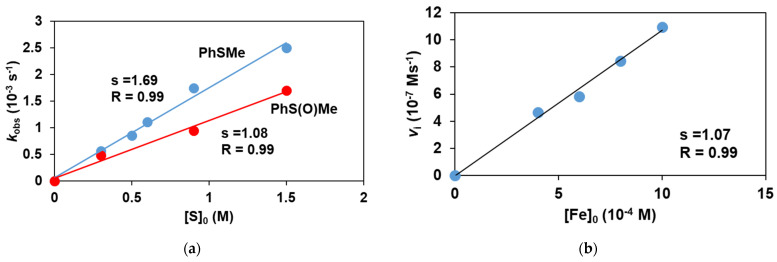
Stoichiometric oxidation of thioanisole and methyl phenyl sulfoxide with Fe^III^(OIPh) generated in situ by the reaction of **1** with 4 equivalents of PhIO. (**a**) Dependence of *k*_obs_ on the initial thioanisole and methyl phenyl sulfoxide concentrations, [[Fe^II^(PBI)_3_](OTf)_2_]_0_ = 1 × 10^−3^ M at 293 K in acetonitrile. (**b**) Dependence of *V*_i_ on the initial complex (**1**) contratio, [PhSMe]_0_ = 6 × 10^−1^ M at 293 K.

**Figure 7:**
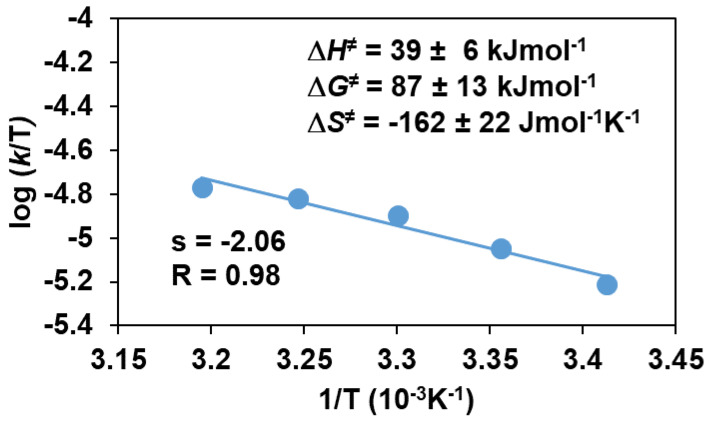
Eyring plot of log*k*/*T* versus 1/*T* for the stoichiometric oxidation of thioanisole with Fe^III^(OIPh) generated in situ by the reaction of **1** with 4 equivalents of PhIO in acetonitrile.

To obtain direct evidence for the involvement of the iron(III)-iodosylbenzene adduct in the sulfoxidation reaction, the stoichiometric reaction of the in situ generated Fe^III^(OIPh) with various thioanisoles was investigated. The Fe^III^(OIPh) species was generated with 4 equivalent of PhIO, and the excess of oxidant was removed by filtration. It was found that this intermediate is able to selectively oxidize the thioanisole derivatives, resulting in an almost quantitative formation of the corresponding sulfoxides suggesting a direct O-atom transfer mechanism with two-electron process. The pseudo-first-order rate constants, *k*_obs_, in the reaction of Fe^III^(OIPh) with thioanisoles were determined from the absorbance change at 760 nm (Figure 5) by monitoring the decrease of the Fe^III^(OIPh) concentration. The yields of sulfoxides without a significant amount of sulfone formation (~1%) were about 80–90%, indicating that the UV-Vis spectral change corresponds to the OAT process. An isobestic point was observed at ~520 nm, indicating that there were no long-lived intermediates during the oxidation reaction (Figure 5a). The kinetic data show that the reaction is first-order in the oxidant, Fe^III^(OIPh) (Figure 5b and Figure 6b), and thioanisole (Figure 6a). The rates in the presence of excess of thioanisole (300–1500 equivalent) obeyed pseudo-first-order kinetics, and the pseudo-first-order rate constants (normalized with the rate of self-decay process without substrates) increased linearly with the thioanisole concentration (Figure 6a). The second-order constants, *k*_2_ were evaluated from the slope of the plots of *k*_obs_ versus [thioanisole]_0_. From this linear plotting, the second-order rate constant (*k*_2_) was determined to be 1.69 × 10^−3^ M^−1^ s^−1^ with *E*_A_ = 41.93 ± 4.45 kJ mol^−1^, Δ*H*^≠^ = 39.41 ± 6.53 kJ mol^−1^, Δ*S*^≠^ = −162.15 ± 21.8 J mol^−1^ K^−1^, Δ*G*^≠^ = 86.92 ± 12.91 kJ mol^−1^ at 293 K (Figure 7). The obtained value is 2 orders of magnitude smaller than those obtained for Fe^IV^(O) species with pentadentate poly-pirydil ligands (*k*_2_ = 2.56 × 10^−1^ M^−1^ s^−1^ with Δ*H*^≠^ = 44 kJ mol^−1^, and Δ*S*^≠^ = −100 J mol^−1^ K^−1^ at 298 K) [12]. A *k*_rel_(PhS(O)Me/PhSMe) value of 0.64 was also determined by comparing an individual reaction with methyl-phenyl-sulfoxide as substrate, with the PhSMe oxidation under identical conditions (Figure 6a). The *k*_obs_ and *k*_2_ values are listed in Table 2.

**Scheme 1:**
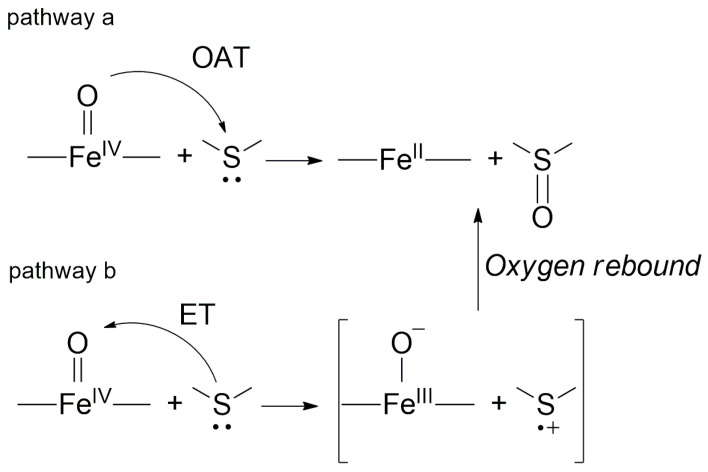
Alternative mechanisms for the oxo-iron-mediated sulfoxidation reactions.

In general, two alternative mechanisms can be proposed for the sulfoxidation of sulfides with oxo-metal complexes, namely direct oxygen atom transfer (OAT) to afford the sulfoxide (pathway a in Scheme 1), and the formation of sulfenium radical via electron transfer (ET) mechanism (pathway b in Scheme 1) [12].

**Figure 8:**
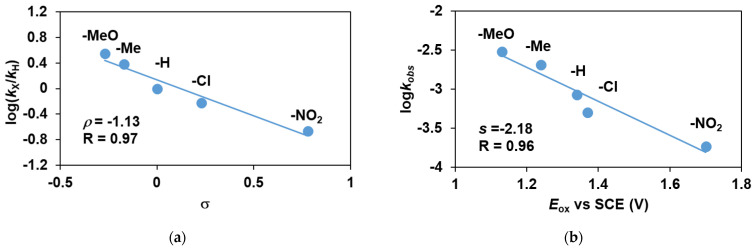
Stoichiometric oxidation of *para*-substituted thioanisole derivatives with Fe^III^(OIPh) generated in situ by the reaction of **1** with 4 equivalents of PhIO in acetonitrile at 293 K: (**a**) Hammett plot of log*k*_rel_ against the *σ*_p_ of para-substituted thioanisoles. (**b**) Plot of log*k*_obs_ against the *E*^0^_ox_ of *para*-substituted thioanisoles. [[Fe^II^(PBI)_3_](OTf)_2_]_0_ = 1 × 10^−3^ M, [4R-PhSMe]_0_ = 5 × 10^−1^ M.

To confirm the mechanism of the reaction as direct OAT rather than electron transfer, we investigated the effect of the substituents in the para position of PhSMe, and the observed kinetic data were treated in terms of the Hammett equation. Electron-attracting substituents in the sulfides reduce the rate of oxidation. The *ρ* value is −1.13, suggesting that the metal-based oxidant is electrophilic (Figure 8a). By plotting log*k*_obs_ against the one-electron oxidation potentials (*E*_ox_) of the thioanisols, a linear line with a slope of −2.18 was obtained, which is consistent with a direct OAT mechanism (Figure 8b). These values are of the same order of magnitude, although slightly smaller than the values obtained for Fe^IV^(O) species with pentadentate poly-pirydil ligands (*ρ* value is −1.78 and −1.51, respectively), indicating the less electrophilic nature of the Fe^III^(OIPh) species compared to the oxo-iron(IV) intermediates with similar ligand environment [12,32]. Gradients of much larger magnitude (−8.5 and −10.5) were observed when reactions proceeded through an ET pathway [33].

**Figure 9:**
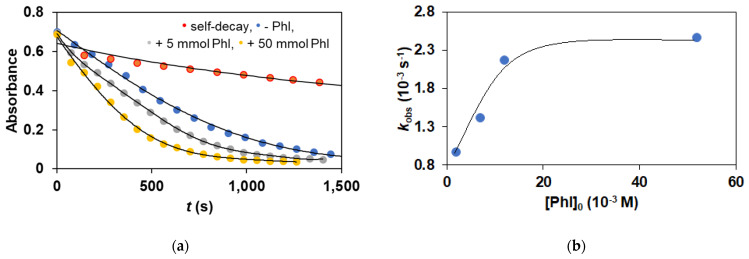
Stoichiometric oxidation of thioanisole with Fe^III^(OIPh) generated in situ by the reaction of **1** with 4 equivalents of PhIO. (**a**) Time course of the oxidation of PhSMe monitored at 760 nm in the absence and in the presence of PhI. (**b**) Dependence of *k*_obs_ on the initial PhI concentrations, [[Fe^II^(PBI)_3_](OTf)_2_]_0_ = 0.6 × 10^−3^ M at 293 K in acetonitrile. [PhSMe]_0_ = 6 × 10^−1^ M at 293 K.

**Scheme 2:**
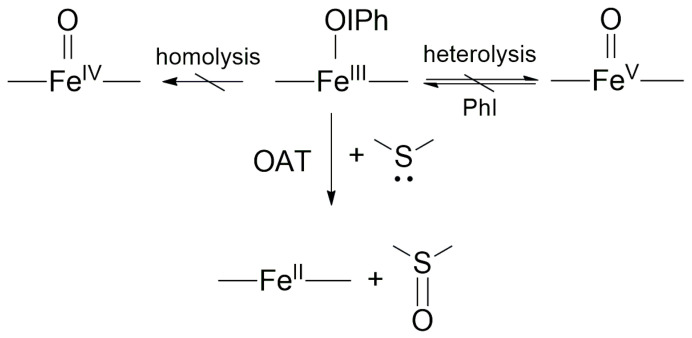
Suggested mechanism for the Fe^III^(OIPh)-mediated sulfoxidation reaction.

Based on these results, we ruled out the participation of the oxo-iron(IV) species, which can be formed by homolytic O-I bond cleavage of iron(III)-iodosyl-benzene adducts, as a reactive intermediate in our stoichiometric and catalytic sulfoxidation reactions. However, we cannot rule out the possibility that the oxo-iron(V) species is formed via heterolytic cleavage of the O-I bond of the iron(III)-iodosyl-benzene adducts in a pre-equilibrium process, and this intermediate participates as a reactive element in the oxidation process. In iron porphyrin systems, an equilibrium exists between an iron(IV) oxo porphyrin π-cation radical complex and an iron(III) iodosylarene porphyrin complex in the presence of iodoarene and it is the iron(IV) center which is the reactive species in olefin epoxidation reactions [19]. If there is an equilibrium between an iron(III)–iodosylarene adduct and an iron(V) oxo species, which is formed by OI bond cleavage of the iron(III)–iodosylarene adduct, the presence of an excess amount of iodoarene should decrease the reaction rate. Compound Fe^III^(OIPh) was employed in the thioanisole oxidation reactions in the presence of PhI (50 equivalent). It was found that the reaction rates were significantly higher than those obtained in the reactions carried out in the absence of PhI (Figure 9). These results demonstrate unambiguously that the iron(III) iodosylarene adduct (Scheme 2), not the iron(V)oxo complex, is the reactive species for the sulfoxidation reactions. When the stoichiometric reaction is carried out with an excess of PhI, a nearly three-fold reaction rate (*k*_obs_ = 2.459 × 10^−3^ s^−1^ (with 50 mmol PhI) compared to *k*_obs_ = 0.97 × 10^−3^ s^−1^ (without PhI)) is observed, suggesting that the Fe^III^(IOPh)-mediated reaction is more favored compared to the oxo-iron(V) species (Figure 9a). The saturation curve clearly shows that the equilibrium is shifted towards the Fe^IlI^(OIPh) species (Figure 9b).

The obtained results are consistent with the results obtained for our catalytic system, based on which the substituent effect tests indicate a direct O-atom transfer from the Fe^III^(OIPh) intermediate to the substrate in the rate-determining step of both systems (Scheme 2). The mechanism of direct oxygen atom transfer mediated by Fe^III^(IOPh) and its differences compared to the ET mechanism were also supported by detailed DFT calculations [21].

### 2.3. Catalytic Epoxidation Reaction Mediated by In Situ Generated Iron(III)-Iodosylbenzene Adduct

**Figure 10:**
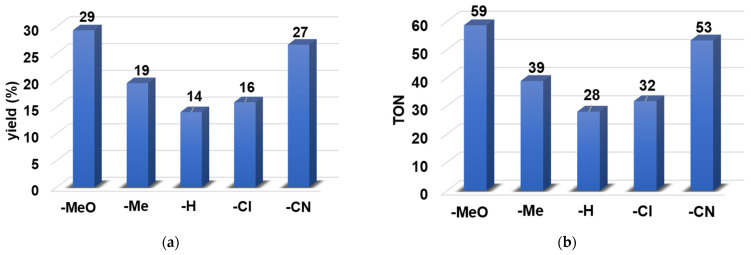
Catalytic oxidation of *para*-substituted styrene derivatives carried out by **1** with PhIO as co-oxidant in acetonitrile at 293 K: (**a**) Reaction yields for para-substituted styrenes. (**b**) TON and TOF values for para-substituted styrenes. [[Fe^II^(PBI)_3_](OTf)_2_]_0_ = 1 × 10^−3^ M, [PhIO]_0_ = 1 × 10^−1^ M, [4R-PhCHCH_2_]_0_ = 3 × 10^−1^ M.

Similarly to the catalytic sulfoxidation reaction, the catalytic activity of the [Fe^II^(PBI)_3_](OTf)_2_ (**1**) was also investigated in the oxidation of para-substituted styrene derivatives, utilizing PhIO as oxidant. The reactions were carried out under standard, optimal catalytic conditions (1:100:300 ratio for catalyst:oxidant:substrate) in acetonitrile at 293 K. The large excess of substrate was used to minimize over-oxidation of the product. It took around 1 h to have about 14–29% yields (based on the oxidant) depending on the substituent on the phenyl ring (Table 3). The iron(II)-catalyzed reactions of styrenes produced epoxide derivatives as expected, and as a major product in all cases, in addition to one minor product, namely benzaldehyde derivatives (less than 1%). The effect of electron-donating and electron-withdrawing substituents on the aromatic ring on the relative reactivity has also been studied and showed a significant impact on the catalytic epoxidation reaction (Table 1 and Figure 10). Styrenes with both electron-donating (-OMe or -Me) and electron-withdrawing (-Cl or -CN) groups on the phenyl ring gave better yields (29% for -OMe, 19% for -Me, and 16% for -Cl and 27% for -CN) (Figure 10a). The highest TON (TOF) values can be observed for 4-OMe-PhCHCH_2_; 59 (59 h^−1^) (Figure 10b).

**Figure 11:**
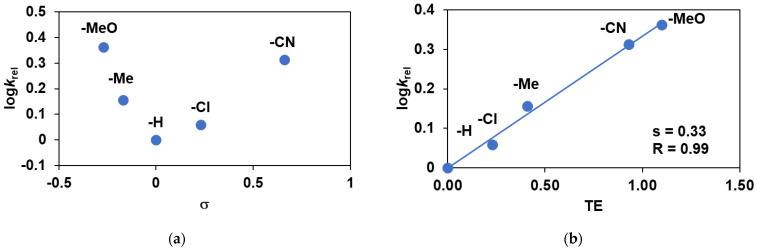
Catalytic oxidation of *para*-substituted styrene derivatives carried out by **1** with PhIO as co-oxidant in acetonitrile at 293 K: (**a**) Hammett plot of log*k*_rel_ against the *σ*_p_ of para-substituted styrenes. (**b**) Plot of log*k*_rel_ against the TE of *para*-substituted styrenes. [[Fe^II^(PBI)_3_](OTf)_2_]_0_ = 1 × 10^−3^ M, [PhIO]_0_ = 1 × 10^−1^ M, [4R-PhCHCH_2_]_0_ = 3 × 10^−1^ M.

**Figure 12:**
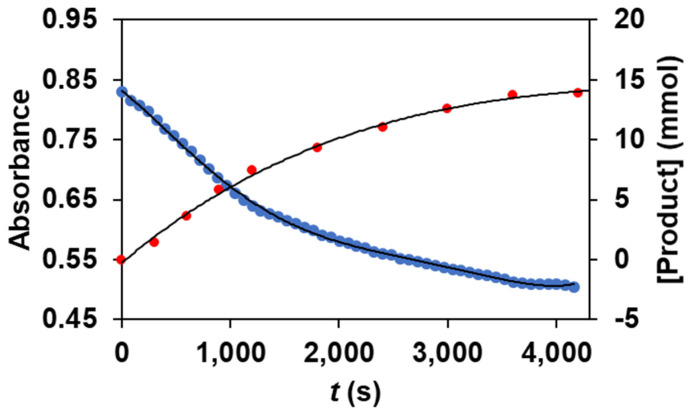
Time course for the catalytic oxidation of styrene (•) carried out by **1** with PhIO as co-oxidant in acetonitrile at 293 K, monitored at 760 nm by UV-Vis and by GC measuring the amounts of the forming styrene oxide (•). [[Fe^II^(PBI)_3_](OTf)_2_]_0_ = 1 × 10^−3^ M, [PhIO]_0_ = 1 × 10^−1^ M, [PhCHCH_2_]_0_ = 3 × 10^−1^ M.

The reactivities of *para*-substituted 4R-PhCHCH_2_ relative to that of PhCHCH_2_ were also investigated (Figure 10 and Figure 11). It was found that styrenes displayed Hammett treatments of relative reactivities (*k*_rel_ = log(*X*_f_/*X*_i_)/log(*Y*_f_/*Y*_i_), where *X*_i_ and *X*_f_ are the initial and final concentration of 4R-PhCHCH_2_, and *Y*_i_ and *Y*_f_ are the initial and final concentration of PhCHCH_2_) of various substituents against *σ* gave concave Hammett curve, which means that both electron-donating and electron-withdrawing groups on the para-position of the aromatic ring increase the rate of the epoxidation reaction (Figure 11a). A linear free-energy relationship between the relative rate (log*k*_rel_) for the para-substituted styrene oxidations and the total substituent effect (TE) parameters has been established: *ρ*_TE_• = +0.33 (Figure 11b) [33]. Based on the obtained results, it can be assumed that the oxidation of aromatic alkenes catalyzed by **1** with PhIO as oxidant takes place through the formation of a rate-limiting radical intermediate. The profile of the catalytic process shows the immediate formation and subsequent decomposition of the spectroscopically characterized Fe^III^(OIPh) species, as well as the formation of the oxidation products that appear parallel to it (Figure 12).

### 2.4. Stoichiometric Epoxidation Reaction Mediated by In Situ Generated Iron(III)-Iodosylbenzene Adduct

**Figure 13:**
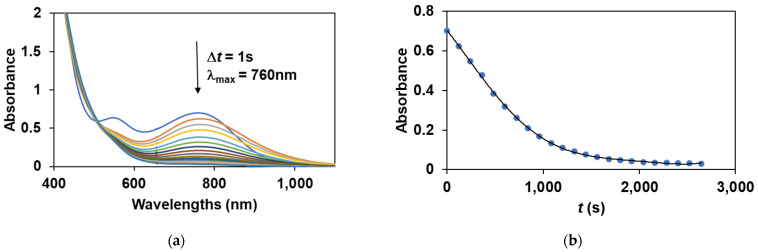
Stoichiometric oxidation of styrene with Fe^III^(OIPh) generated in situ by the reaction of **1** with 4 equivalents of PhIO. (**a**) UV-Vis spectral change of Fe^III^(OIPh) (0.4 mM) in the presence of PhCHCH_2_ (60 mM). (**b**) Time course of the oxidation of PhCHCH_2_ monitored at 760 nm (**a**).

**Figure 14:**
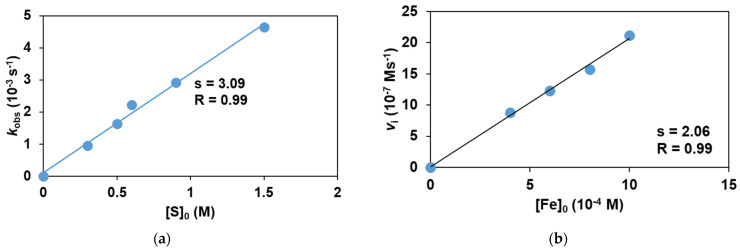
Stoichiometric oxidation of styrene with Fe^III^(OIPh) generated in situ by the reaction of **1** with 4 equivalents of PhIO. (**a**) Dependence of *k*_obs_ on the initial concentrations, [[Fe^II^(PBI)_3_](OTf)_2_]_0_ = 1 × 10^−3^ M at 293 K in acetonitrile. (**b**) Dependence of *V*_i_ on the initial complex (**1**) contration, [PhCHCH_2_]_0_ = 6 × 10^−1^ M at 293 K.

The epoxidation of styrene derivatives with **1** and PhIO as an oxidant was carried out at room temperature under catalytic reaction conditions, and the corresponding epoxides were obtained as the main products in these reactions. Since the product distributions were identical to those observed in the stoichiometric reactions with in situ generated iron(III)-iodosylbenzene adduct, this lead us to suggest that the active oxidizing species responsible for the olefin epoxidation in these catalytic reactions is the iron(III)-iodosylbenzene adduct (Figure 12). In order to clarify the elementary step of the catalytic reaction, we performed detailed reaction kinetic measurements. The reactivity of the in situ generated iron(III)-iodosylbenzene was investigated in the epoxidation reaction of styrene derivatives. The Fe^III^(OIPh) intermediate was generated by the reaction of **1** with 1.2 equivalent of PhIO, and the rate of its decay at 760 nm was measured as a function of the concentration of added styrene derivatives (Figure 13). An isobestic point was observed at ~520 nm, indicating that there were no long-lived intermediates during the oxidation reaction (Figure 13a). The rates in the presence of a large excess of styrene (300–1500 equiv.) obeyed pseudo-first order kinetics −d[Fe^III^(OIPh)]/d*t* = *k*_obs_[Fe^III^(OIPh)], where *k*_obs_ = *k*_sd_ + *k*_2_[S] and *k*_sd_ << *k*_2_[PhCHCH_2_]), which is evident from the linear log(absorbance) versus time plots (Figure 13b), and the pseudo-first order rate constants (*k*_obs_ = *k*_2_[PhCHCH_2_)] increased proportionally with the substrate concentration (Figure 14a). At constant styrene concentration the linear plot of the reaction rate values (*V*_i_ = *k*_obs_[Fe^III^(OIPh)]) against the initial concentration of Fe^III^(OIPh) states that the reaction is first-order with respect to the Fe^III^(OIPh) concentration (Figure 14b).

**Figure 15:**
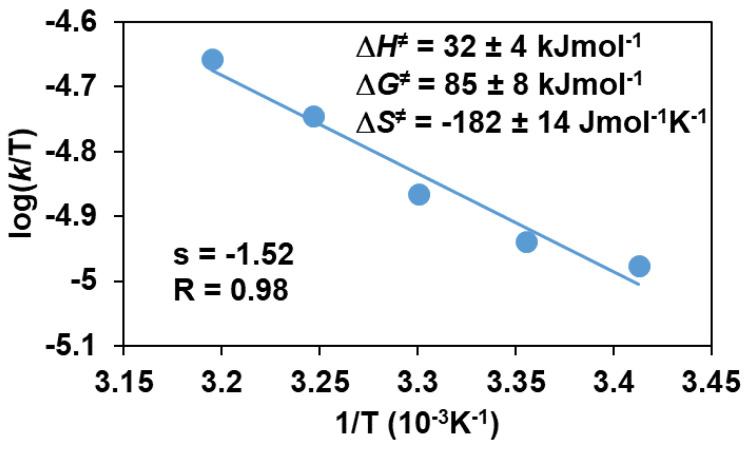
Eyring plot of log*k*/*T* versus 1/*T* for the stoichiometric oxidation of styrene with Fe^III^(OIPh) generated in situ by the reaction of **1** with 4 equivalents of PhIO in acetonitrile.

The above results establish a rate law of −d[Fe^III^(OIPh)]/d*t* = *k*_2_[Fe^III^(OIPh)][PhCHCH_2_] for the styrene derivatives. From these experiments, the second order rate constant (*k*_2_) was determined to be 3.09 × 10^−3^ M^−1^ s^−1^ with Δ*H*^≠^ = 32 ± 4 kJ mol^−1^, Δ*S*^≠^ = −182 ± 14 J mol^−1^ K^−1^, Δ*G*^≠^ = 85 ± 8 kJ mol^−1^ at 293 K (Table 4 and Figure 15). This value is an order of magnitude larger than that was found for Fe^IV^(O) species with pentadentate poly-pirydil ligands (*k*_2_ = 2.94 × 10^−4^ M^−1^ s^−1^ with Δ*G*^≠^ = 93 ± 8 kJ mol^−1^ at 298 K) [34]. Comparing the obtained values with the values calculated for sulfoxidation, it can be concluded that the epoxidation is slightly more favorable, approximately twice as fast, if the reactions are carried out under the same conditions. The lower ΔG^≠^ value obtained for epoxidation is also consistent with the above result. The relatively large and negative entropies are typical of associative processes.

**Figure 16:**
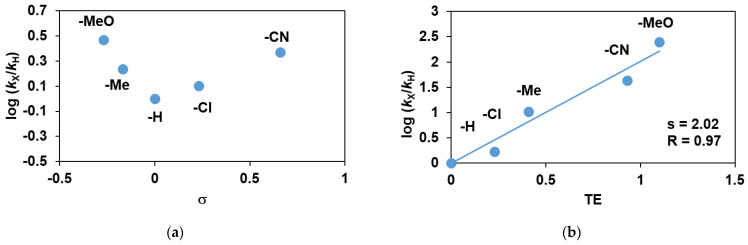
Stoichiometric oxidation of *para*-substituted styrene derivatives with Fe^III^(OIPh) generated in situ by the reaction of **1** with 4 equivalents of PhIO in acetonitrile at 293 K: (**a**) Hammett plot of log*k*_rel_ against the *σ*_p_ of *para*-substituted styrenes. (**b**) Plot of log*k*_rel_ against the TE of para-substituted styrenes. [[Fe^II^(PBI)_3_](OTf)_2_]_0_ = 1 × 10^−3^ M, [4R-PhCHCH_2_]_0_ = 3 × 10^−1^ M.

Competitive reactions were also performed with para-substituted styrene derivatives to evaluate the effect of electronic factors on the reaction. Since both electron-donating (-OMe, -Me) and electron-withdrawing (-Cl, -CN) substituents can accelerate the reaction, the correlation of relative reactivity (log *k*_rel_) on the substituent constants (σ+) of para-substituted styrenes is not linear, resulting in a concave Hammett curve (16a) [13]. This result is in contrast to the styrene oxidations by oxo-iron(IV) systems, wherein typically linear Hammett correlations of log*k*_rel_ versus σ+ with *ρ*+ of ~−2.0 were obtained [14,34]. A linear free-energy relationship was established with *ρ*_TE_• = +2.02 value between the relative rates (log*k*_rel_) of *para*-substituted styrene oxidations and the total substituent effect (TE, stabilities of the benzylic radicals including spin delocalization and polar effects (Figure 16b) [33]).

**Scheme 3:**
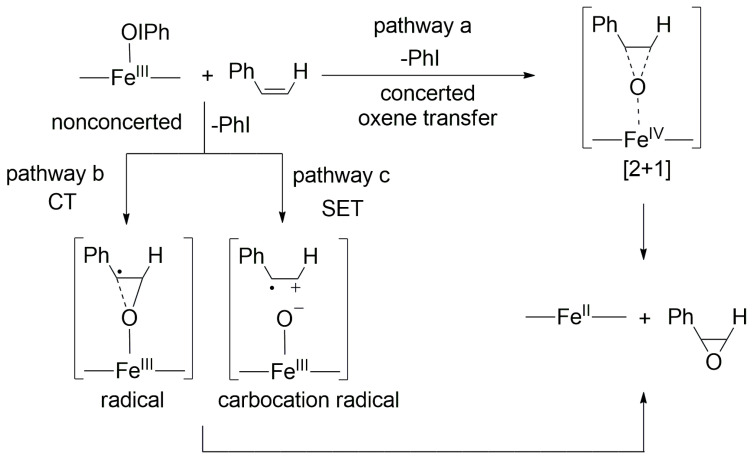
Alternative mechanisms for the Fe^III^(OIPh)-mediated epoxidation reactions.

In general, three alternative mechanisms, including three possible intermediates, can be proposed for the epoxidation of alkenes with oxo-metal complexes, namely one concerted via [2 + 1] oxene insertion (pathway a in Scheme 3), and two nonconcerted via an alkene-derived benzylic radical (pathway b in Scheme 3) or a carbocation intermediate (pathway c in Scheme 3).

When the stoichiometric reaction is carried out with an excess of PhI, no inhibition was observed, suggesting that the Fe^III^(IOPh)-mediated reaction is more favored compared to the oxo-iron(V) species. The sensitivity of the *k*_2_ values to the para-substituent effect and the concave Hammett curve support the radical mechanism, including the rate-limiting formation of benzyl radical species. If the carbocation ion is the assumed intermediate, a more negative *ρ*^+^ value is expected in the electrophilic process (~−3.5). The benzylic radical formed then undergoes ring closure to form an epoxide. Since a similar trend and behavior was observed during the catalytic and stoichiometric reactions, the reaction mediated by Fe^III^(IOPh) intermediate can be considered as an elementary step in the catalytic process.

## 3. Experimental

### 3.1. Materials and Methods

All chemicals including PBI ligands and substrates were obtained from Aldrich Chemical Co. and used without further purification unless otherwise indicated. Solvents were dried according to published procedures and distilled, stored under argon [35]. [Fe(PBI)_3_](CF_3_SO_3_)_2_ (**1**) was synthesized according to literature methods [28]. Iodosylbenzene (PhIO) was prepared by literature methods [36]. UV-visible spectra were recorded on an Agilent 8453 diode-array spectrophotometer using quartz cells. IR spectra were recorded using a Thermo Nicolet Avatar 330 FT-IR instrument (Thermo Nicolet Corporation, Madison, WI, USA). Samples were prepared in the form of KBr pellets. GC analyses were performed on an Agilent 6850 (Budapest, Hungary) gas chromatograph equipped with a flame ionization detector and a 30 m HP-5MS column. GC-MS analyses were carried out on a Shimadzu QP2010SE (Budapest, Hungary) equipped with a secondary electron multiplier detector with conversion dynode and a 30 m HP5MS column.

### 3.2. Reactivity Studies and Product Analysis

All stoichiometric reactions were run in a 1.0 cm UV cuvette and followed by monitoring UV-vis spectral changes in the reaction solutions, rate constants were determined under pseudo-first-order conditions (e.g., [substrate]/[Fe^III^(OIPh)] > 10) by fitting the absorbance changes at 760 nm in CH_3_CN at 293–313 K, respectively, and the resulting solutions were used directly in reactions with substrates, such as thioanisoles, and styrenes, for oxygenation reactions. Reactions were run at least in triplicate, and the data reported are the average of the reactions.

*Stoichiometric reactions*: In a typical reaction [Fe^II^(PBI)_3_](OTf)_2_ (1 × 10^−3^ M) was dissolved in acetonitrile (2 mL) and iodosylbenzene (4 × 10^−3^ M) was added to the solution. The mixture was stirred for 4 min and then the excess of iodosylbenzene was removed by filtration. Substrate (0.3–1.5 M) was added to the solution and the reaction was monitored with a UV-Vis spectrophotometer at 760 nm. The product was identified by GC-MS and the yields were determined by GC using bromobenzene as internal standard.

*Catalytic reactions*: [Fe^II^(PBI)_3_](OTf)_2_ (1 × 10^−3^ M) was dissolved in acetonitrile (3 mL), and iodosylbenzene (1 × 10^−1^ M) and substrate (3–5 × 10^−1^ M) was added to the solution. The reaction was monitored by UV-Vis spectroscopy at 293 K in a cuvette. These reactions were also followed by GC: [Fe^II^(PBI)_3_](OTf)_2_ (1 × 10^−3^ M) was dissolved in acetonitrile (10 mL), and PhIO (1 × 10^−1^ M) and substrate (3–5 × 10^−1^ M) were added to the solution. Bromobenzene (1 × 10^−1^ M) was added to the solution as an internal standard. The mixture was stirred for 1 h and, during this time, the product yields were determined every 10 min by GC.

Products of substituted thioanisole oxidation:

Thioanisole/methyl phenyl sulfoxide: (*m/z*) relative intensity 140 (M^+^, 77.02); 124.95 (100); 97 (86.14); 94 (22.79); 91 (10.05); 77 (70.10); 74 (10.88); 65 (22.7); 53 (15.7); 51 (75.36); 50 (30.96);45 (16.48); 39 (16.69)/methyl phenyl sulfone: (*m*/*z*) relative intensity 155.95 (M^+^, 12.62); 141 (15.54); 94 (29.93); 77 (100); 51 (50.10); 50 (18.33); 39.05 (8.89).

4-metoxythioanisole/p-methoxyphenyl methyl sulfoxide: (*m/z*) relative intensity 170 (M^+^, 48.93); 154.95 (100); 139 (34.61); 103.95 (25.71); 90 (37.52); 89 (15.75); 77 (36.95); 67.05 (24.86); 65 (38.52); 63 (19.43); 51 (10.86); 45 (16.54); 39.05 (28.97)/p-methoxyphenyl methyl sulfone: (m/z) relative intensity 186.2 (M^+^, 18.53);171 (36.36); 155 (18.52); 139 (15.61); 106.9 (28.90); 92 (14.54); 91.05 (58.18); 89.1 (17.26); 79 (18.92); 77 (100); 65.05 (40.92); 63 (18.93); 51.1 (16.33); 39 (37.54).

Methyl p-tolyl sulfide/methyl p-tolyl sulfoxide: (*m/z*) relative intensity 153.95 (M^+^, 50.80); 139 (100); 111 (17.52); 108 (12.39); 90 (36.92); 89 (13.38); 79 (10.70); 78 (14.96); 77 (43.63); 67.05 (22.84); 65 (35.81); 63 (21.10); 51 (11.45); 45 (14.57); 39.05 (25.46)/methyl p-tolyl sulfone: (*m*/*z*) relative intensity 170.05 (M^+^, 15.08); 154.9 (16.37); 139.1 (15.61); 107.1 (23.60); 92.2 (11.44); 91.05 (100); 89.1 (12.26); 79 (14.09); 77 (23.1); 65.05 (45.96); 63 (20.24); 51.1 (13.66); 39.05 (34.54).

4-chlorothioanisole/p-chlorophenyl methyl sulfoxide: (*m/z*) relative intensity 175.9 (M^+^, 17.26); 17.9 (49.04); 160.9 (37.02); 158.9 (100); 132.95 (21.51); 130.95 (58.38); 128 (18.89); 126.95 (12.67); 110.95 (32.58); 108 (13.97); 98.95 (15.1); 77 (5.29); 76 (11.74); 75 (65.71); 74 (25.12); 73 (11.39); 63 (18.47); 51.05 (15.50); 50.05 (36.25); 45 (18.56)/p-chlorophenyl methyl sulfone: (*m*/*z*) relative intensity 190 (M^+^, 11.60); 175 (19.60); 129 (11.20); 128 (13.20); 127 (31.60); 113 (32.4); 112 (8.4); 111 (100); 99 (15.6); 77 (8.16); 76 (11.2); 75 (59.2); 74 (23.2); 73 (9.2); 51 (11.2); 50 (31.2).

4-nitrothioanisole/p-nitrophenyl methyl sulfoxide: (*m/z*) relative intensity 185.9 (M^+^, 9.76); 184.9 (100); 169.95 (51.51); 139.95 (33.51); 139 (10.81); 124 (26.41); 123 (7.85); 112 (34.61); 111 (16.21); 110 (11.17); 108 (12.88); 96 (28.54); 95 (17.13); 92 (20.43); 82.05 (19.09); 77 (20.14); 76 (47.21); 75 (33.45); 74 (31.63); 70 (30.94); 68.95 (21.28); 65.05 (13.62); 64 (22.02); 63 (44.94); 62.05 (10.33); 58 (12.09); 51 (20.36); 50 (78.27); 45 (32.47); 39 (16.16)/p-nitrophenyl methyl sulfone: (*m*/*z*) relative intensity 201.9 (M^+^, 12.8); 201 (100); 186 (31.21); 156 (21.56); 155 (12.31); 139.9 (17.41); 139 (11.35); 128 (43.81); 137 (14.71); 125.95 (13.27); 124 (14.93); 112 (25.84); 111 (21.13); 108 (26.03); 97.95 (18.52); 93 (18.54); 77 (17.14); 76 (38.21); 75 (28.57); 74 (35.39); 70 (26.37); 69 (23.87); 65 (17.58); 63.95 (24.72); 63 (40.85); 62 (11.73); 58 (16.82); 51 (19.43); 50 (77.3); 45 (31.96); 39 (14.54).

Products of substituted styrene oxidation:

Styrene/styrene oxide: (*m*/*z*) relative intensity 120.05 (M^+^, 24.05); 119 (45.13); 92 (43.84); 91.05 (100); 90.05 (61.15); 89 (70.51); 77.1 (19.47); 73.8 (8.24); 65 (25.7); 64 (10.72); 63 (33.95); 62.05 (9.73); 51.05 (32.36);50.05 (27.81); 41 (10.75); 39 (30.49); 38 (14.5).

4-vinylanisole/2-(4-methoxyphenyl)oxirane: (*m*/*z*) relative intensity 150.1 (M^+^, 33.25); 135 (74.19); 119.05 (57.95); 92 (37.14); 90.95 (100); 90 (58.91); 89.05 (66.43); 77 (27.93); 74 (10.86); 65 (31.8); 64 (13.64); 63 (36.64); 62 (10.65); 51.05 (28.54); 50.05 (24.87); 41 (15.85); 39 (35.85); 38 (12.64).

4-methylstyrene/2-(4-methylphenyl)oxirane: (*m/z*) relative intensity 134.1 (M^+^, 28.53); 119 (100); 91.95 (47.32); 91 (63.29); 90.05 (57.05); 89.05 (64.64); 77 (27.92); 74.05 (10.74); 64.95 (23.95); 64 (14.92); 63.05 (36.17); 62 (15.96); 51.05 (28.95);50.05 (35.17); 41.05 (13.47); 39.05 (34.75); 38 (17.94).

4-chlorostyrene/2-(4-chlorophenyl)oxirane: (*m/z*) relative intensity 154.1 (M^+^, 36.86); 119.05 (37.75); 92 (47.98); 91 (100); 90.05 (56.91); 89 (69.43); 77.1 (23.97); 74 (11.52); 65.05 (28.93); 64.05 (15.72); 63 (38.91); 62 (13.84); 51 (37.47);50.05 (36.81); 41.05 (14.74); 39 (31.65); 38.05 (17.43).

4-cianostyrene/2-(4-cianophenyl)oxirane: (*m/z*) relative intensity 145.1 (M^+^, 41.96); 119.05 (100); 91.95 (42.57); 91 (48.24); 90.05 (47.93); 89 (53.96); 77 (29.34); 74 (16.83); 65 (33.83); 64 (12.54); 63 (35.94); 62 (16.83); 51.05 (42.65); 50 (39.75); 41 (18.63); 39 (37.75); 38 (22.83).

## 4. Conclusions

In summary, we reported the catalytic oxidation of thioanisole and styrene derivatives using a non-heme complex, Fe^II^(PBI)_3_ as catalyst and PhIO as oxidant. The catalytic activity was moderate, but proved to be selective for both substrates, producing sulfoxides and epoxides, respectively, as the main products. Based on Hammett correlation for *para*-substituted thioanisoles, electrophilic iron-based oxidation can be assumed via a direct O-atom transfer mechanism. In contrast, in the case of para-substituted styrenes, a nonconcerted electron transfer mechanism can be proposed through the formation of the radicaloid benzylic radical intermediate in the rate-determining step. The results of the stoichiometric sulfoxidation and epoxidation reactions of the in situ generated Fe^III^(OIPh) intermediate supported our proposed hypotheses for the investigated catalytic reactions. These results provide further example of metal–oxidant adducts which can transfer their oxygen atom to organic substrates prior to the conversion into metal oxo species. Efforts to develop asymmetric catalyzed oxidation of various alkenes and sulfides of higher technological interest by introducing the chiral moiety to ligands or oxidants as well as their detailed mechanistic aspects are under progress. Future studies will also be focused on elucidating the intriguing reactivity differences between high- and low-spin Fe(III)-iodosylarene complexes. This type of reactivity difference has been demonstrated recently in the reactions of high- and low-spin iron(III)-hydroperoxo and high-valent oxoiron complexes.

## Figures and Tables

**Table 1:** Catalytic oxidation of *para*-substituted thioanisole derivatives carried out by **1** with PhIO as co-oxidant in acetonitrile at 293 K ^1^.

Entry	4R-PhSCH_3_	*E*_ox_ vs. SCE (V)	*σ*_p_	Yield (%) ^2^	TON ^3^	TOF (h^−1^) ^4^
**1**	H	1.34	0	52 (1.8)	52	63
**2**	OMe	1.13	−0.27	66 (2.3)	66	80
**3**	Me	1.24	−0.17	60 (2.1)	60	72
**4**	Cl	1.37	0.23	37 (1.8)	37	45
**5**	NO_2_	1.70	0.78	21 (0.0)	21	25

^1^ Reaction conditions: See Section 3. ^2^ The product was identified by GC-MS and the yields (based on oxidant) were determined by GC using bromobenzene as internal standard. ^3^ [Substrate]_0_/[1]_0_. ^4^ TON/h.

**Table 2:** Kinetic data for the stoichiometric oxidation of *para*-substituted thioanisole and methyl phenyl sulfoxide derivatives with Fe^III^(OIPh) generated in situ by the reaction of **1** with 4 equivalents of PhIO in acetonitrile ^1^.

Entry	[1] (mM)	4R-PhSMe (M)	T (K)	*σ*_p_ (4R)	*E_ox_* vs. *SCE* (V)	*V*_i_ (10^−6^ Ms^−1^) ^1^	*k*_obs_’ (10^−3^ s^−1^) ^2^	*k*_2_ (10^−3^ M^−1^ s^−1^) ^1^
**1**	0.4	0.6	293	0 (H)	1.34	0.46	1.12	1.90
**2**	0.6	0.6	293	0 (H)	1.34	0.58	0.97	1.62
**3**	0.6	0.6	293	0 (H)	1.34		1.41 ^3^	
**4**	0.6	0.6	293	0 (H)	1.34		2.17 ^4^	
**5**	0.6	0.6	293	0 (H)	1.34		2.46 ^5^	
**6**	0.8	0.6	293	0 (H)	1.34	0.85	1.05	1.76
**7**	1.0	0.6	293	0 (H)	1.34	1.10	1.09	1.82
**8**	1.0	0.3	293	0 (H)	1.34		0.56	1.87
**9**	1.0	0.5	293	0 (H)	1.34		0.85	1.70
**10**	1.0	0.9	293	0 (H)	1.34		1.75	1.94
**11**	1.0	1.5	293	0 (H)	1.34		2.49	1.66
**12**	1.0	1.5	298	0 (H)	1.34		4.01	2.67
**13**	1.0	1.5	303	0 (H)	1.34		5.78	3.86
**14**	1.0	1.5	308	0 (H)	1.34		6.98	4.65
**15**	1.0	1.5	313	0 (H)	1.34		8.03	5.35
**16**	1.0	0.5	293	−0.27 (OMe)	1.13		3.22	6.03
**17**	1.0	0.5	293	−0.17 (Me)	1.24		2.25	4.09
**18**	1.0	0.5	293	0.23 (Cl)	1.37		0.51	1.01
**19**	1.0	0.5	293	0.78 (NO_2_)	1.70		0.18	0.37
**20**	1.0	0.3	293				0.48	1.59
**21**	1.0	0.9	293				0.94	1.05
**22**	1.0	1.5	293				1.69	1.13

^1^ Reaction conditions: See Section 3. ^2^ *k*_obs_’ = *k*_obs_ *− k*_sd_. ^3^ In the presence of 5 mmol PhI. ^4^ In the presence of 10 mmol PhI. ^5^ In the presence of 50 mmol PhI.

**Table 3:** Catalytic oxidation of *para*-substituted styrene derivatives carried out by **1** with PhIO as co-oxidant in acetonitrile at 293 K ^1^.

Entry	4R-PhCHCH_2_	TE ^5^	*σ*_p_	Yield (%) ^2^	TON ^3^	TOF (h^−1^) ^4^
**1**	H	0.00	0	14	28	28
**2**	OMe	1.10	−0.27	29	59	59
**3**	Me	0.41	−0.17	19	39	39
**4**	Cl	0.23	0.23	16	32	32
**5**	CN	0.93	0.66	27	53	53

^1^ Reaction conditions: See Section 3. ^2^ The product was identified by GC-MS and the yields (based on oxidant) were determined by GC using bromobenzene as internal standard. ^3^ [Substrate]_0_/[1]_0_. ^4^ TON/h. ^5^ TE = total substituent effect [33].

**Table 4:** Kinetic data for the stoichiometric oxidation of *para*-substituted styrenes with Fe^III^(OIPh) generated in situ by the reaction of **1** with 4 equivalents of PhIO in acetonitrile ^1^.

Entry	[1] (mM)	4R-PhCHCH_2_ (M)	T (K)	*σ*_p_ (4R)	TE ^3^	*V*_i_ (10^−6^ Ms^−1^)	*k*_obs_’ (10^−3^ s^−1^)	*k*_2_ (10^−3^ M^−1^ s^−1^) ^2^
**1**	0.4	0.6	293	0 (H)	0	0.88	2.20	3.67
**2**	0.6	0.6	293	0 (H)	0	1.23	2.05	3.42
**3**	0.8	0.6	293	0 (H)	0	1.57	1.96	3.27
**4**	1.0	0.6	293	0 (H)	0	2.12	2.12	3.53
**5**	1.0	0.3	293	0 (H)	0		0.95	3.18
**6**	1.0	0.5	293	0 (H)	0		1.64	3.27
**7**	1.0	0.9	293	0 (H)	0		2.92	3.24
**8**	1.0	1.5	293	0 (H)	0		4.64	3.10
**9**	1.0	1.5	298	0 (H)	0		5.14	3.42
**10**	1.0	1.5	303	0 (H)	0		6.18	4.12
**11**	1.0	1.5	308	0 (H)	0		8.29	5.53
**12**	1.0	1.5	313	0 (H)	0		10.3	6.68
**13**	1.0	0.3	293	−0.27 (OMe)	1.10		2.81	9.33
**14**	1.0	0.3	293	−0.17 (Me)	0.41		1.64	5.47
**15**	1.0	0.3	293	0.23 (Cl)	0.23		1.21	4.02
**16**	1.0	0.3	293	0.78 (CN)	0.66		2.23	7.43

^1^ Reaction conditions: See Section 3. ^2^ *k*_obs_ = *k*_obs_’-*k*_sd_. ^3^ TE = total substituent effect [33].

## Data Availability

Not applicable.

## Sample Availability

Samples of the compounds are not available.
